# Dieckol and Its Derivatives as Potential Inhibitors of SARS-CoV-2 Spike Protein (UK Strain: VUI 202012/01): A Computational Study

**DOI:** 10.3390/md19050242

**Published:** 2021-04-25

**Authors:** Mohammad Aatif, Ghazala Muteeb, Abdulrahman Alsultan, Adil Alshoaibi, Bachir Yahia Khelif

**Affiliations:** 1 Department of Public Health, College of Applied Medical Science, King Faisal University, Al-Ahsa 31982, Saudi Arabia; bkhelif@kfu.edu.sa; 2 Department of Nursing, College of Applied Medical Science, King Faisal University, Al-Ahsa 31982, Saudi Arabia; graza@kfu.edu.sa; 3 Department of Biomedical Sciences, College of Applied Medical Science, King Faisal University, Al-Ahsa 31982, Saudi Arabia; aalsultan@kfu.edu.sa; 4 Department of Physics, College of Science, King Faisal University, Al-Ahsa 31982, Saudi Arabia; adshoaibi@kfu.edu.sa

**Keywords:** COVID-19, natural compounds, marine-derived compounds, molecular docking and simulation, seaweeds, spike protein

## Abstract

The high risk of morbidity and mortality associated with SARS-CoV-2 has accelerated the development of many potential vaccines. However, these vaccines are designed against SARS-CoV-2 isolated in Wuhan, China, and thereby may not be effective against other SARS-CoV-2 variants such as the United Kingdom variant (VUI-202012/01). The UK SARS-CoV-2 variant possesses D614G mutation in the Spike protein, which impart it a high rate of infection. Therefore, newer strategies are warranted to design novel vaccines and drug candidates specifically designed against the mutated forms of SARS-CoV-2. One such strategy is to target ACE2 (angiotensin-converting enzyme2)–Spike protein RBD (receptor binding domain) interaction. Here, we generated a homology model of Spike protein RBD of SARS-CoV-2 UK strain and screened a marine seaweed database employing different computational approaches. On the basis of high-throughput virtual screening, standard precision, and extra precision molecular docking, we identified BE011 (Dieckol) as the most potent compounds against RBD. However, Dieckol did not display drug-like properties, and thus different derivatives of it were generated in silico and evaluated for binding potential and drug-like properties. One Dieckol derivative (DK07) displayed good binding affinity for RBD along with acceptable physicochemical, pharmacokinetic, drug-likeness, and ADMET properties. Analysis of the RBD–DK07 interaction suggested the formation of hydrogen bonds, electrostatic interactions, and hydrophobic interactions with key residues mediating the ACE2–RBD interaction. Molecular dynamics simulation confirmed the stability of the RBD–DK07 complex. Free energy calculations suggested the primary role of electrostatic and Van der Waals’ interaction in stabilizing the RBD–DK07 complex. Thus, DK07 may be developed as a potential inhibitor of the RBD–ACE2 interaction. However, these results warrant further validation by in vitro and in vivo studies.

## 1. Introduction

The WHO (World Health Organization) declared the outbreak of SARS-CoV-2 (severe acute respiratory syndrome coronavirus 2) as public health emergency of international concern on 30 January 2020, and a pandemic on 11 Mar 2020, due to the rapid spread of the virus and absence of any preexisting medications [1]. SARS-CoV-2 causes COVID-19, a disease of the respiratory system with symptoms varying from mild flu-like to more severe respiratory disorders characterized by high fever, body ache, tiredness, cough, and difficulty in breathing. In critical cases, patients need hospitalization due to severe pneumonia-like conditions. The first case of COVID-19 was reported in Wuhan (Hubei province, China) in December 2019, and since then it has spread throughout the world [2,3]. As of 17 April 2021, more than 127.3 million people have been affected by COVID-19, with a mortality of around 2.97 million (https://covid19.who.int (accessed on Saturday, 17 April 2021)).

SARS-CoV-2 is a member of *Betacoronavirus* genus in the subfamily *Orthocoronavirinae* in the family *Coronaviridae*, which belongs to *Nidovirales* order [4,5]. It is the seventh known human coronavirus (HCoV) to infect humans, the six others being 229E, OC43, HKU1, NL63, MERS-CoV (Middle East respiratory syndrome coronavirus), and SARS-CoV (severe acute respiratory syndrome coronavirus). Amongst HCoVs, only SARS-CoV, MERS-CoV, and SARS-CoV-2 are highly contagious, pathogenic, and cause serve infection. SARS-CoV-2 harbors around 30 kb (+)-ssRNA encoding 11 open reading frames (ORFs). The 5′ end contains ORFs 1a and 1b, which become translated into pp1a and pp1ab polypeptides, respectively [6]. The 3′ end contains several ORFs encoding structural proteins such as spike protein (S), envelope protein (E), nucleocapsid protein (N), and other accessory proteins [7]. For the normal reproduction and functioning of virus, the single polypeptide chain translated by the virus RNA is further cleaved into structural and non-structural proteins by viral proteases such as PLpro and 3CLpro or Mpro [5]. An essential step of viral infection is, however, the entry of SARS-CoV-2 into host cell, which is mediated by the Spike protein. Spike protein interacts with the cell surface receptor of host cells (ACE2: angiotensin-converting enzyme 2) and facilitates virus entry into the host cell. Thus, Spike protein serves as the most crucial target for developing vaccine or drugs in a fight against COVID-19 [8,9].

Recently, the United Kingdom (UK) faced a surge in COVID-19 cases primarily due to a new strain of SARS-CoV-2 named VUI-202012/01 (variant under investigation, the year 2020, month 12, variant 01). The new variant is characterized by many mutations such as Δ69–70, Δ144, Tyr453Phe, Asn501Tyr, Ala570Asp, Asp614Gly, Phe681His, Thr716Ile, Ser982Ala, and Asp1118His in the spike protein. Of note, Tyr453Phe and Asn501Tyr mutations are located in the receptor-binding domain (RBD) of SARS-CoV-2. Preliminary analyses suggest that this variant has a high rate of transmissibility (up to 70%) with an increased reproductive number (R) compared to other circulating variants.

The genome of SARS-CoV-2 is around 80.0% identical to that of SARS-CoV, thereby suggesting a similar method adopted by the virus to gain entry into the host cell [10]. A mature Spike protein of SARS-CoV-2 is composed of 1273 amino acid residues and comprises a signal peptide (1–13 aa) at the N-terminal end, S1 subunit (14–685 aa), and S2 subunit (686–1273 aa). The S1 subunit can further be categorized into an N-terminal domain (14–305 aa) and a receptor-binding domain (RBD; 319–541 aa), while the S2 subunit can be divided into a fusion peptide (788–806 aa), heptapeptide repeat sequence 1 (HR1; 912–984 aa), HR2 (1163–1213 aa), a transmembrane domain (TM; 1214–1237 aa), and a cytoplasmic domain (CPD; 1238–1273 aa). Similar to SARS-CoV, S1 and S2 subunits of SARS-CoV-2 are responsible for the recognition of ACE2 receptors on the surface of the host and the fusion of the virus membrane with the host membrane, respectively. Human ACE2 is a membrane-bound metallo-carboxypeptidase enzyme primarily responsible for the maturation of human angiotensin (Ang). A full length ACE2 comprises two domains, namely, peptidase domain (PD) located at the N-terminal end and a collectrin-like domain (CLD) at the C-terminal end. The interaction between virus and host cell is mediated by recognizing the ACE2 PD domain by RBD of the S1 subunit of Spike protein. After the RBD-ACE2 interaction, HR1 and HR2 domains in the S2 subunit come close to each other to form a six-helix bundle (6-HB) fusion core, bringing the viral and host cell membranes in close vicinity for fusion and internalization of the virus.

Despite enormous efforts in drug design and development, there is no drug available to treat COVID-19. Several drugs have been considered an option to treat COVID-19, although they have not been explicitly designed against SARS-CoV-2. These include antiviral drugs (Remdesivir, Ritonavir, Lopinavir, Ribavirin, and Fapiravir), Ivermectin, etc. As of Mar 2021, 12 vaccines have been authorized for public use as a preventive measure against COVID-19 by at least one national regulatory authority. These vaccines and their developers are Pfizer-BioNTech (Pfizer and BioNTech, USA and Germany), Johnson & Johnson (Janssen vaccines, USA and Netherlands), Moderna (Moderna, Cambridge, MA, USA), Oxford–AstraZeneca (University of Oxford, Oxford, OX1 2JD, United Kingdom and AstraZeneca, Södertälje, Sweden), Sputnik V (Gamaleya Research Institute of Epidemiology and Microbiology, Russia), BBIBO-CorV (Sinopharm, Chaoyang District, Beijing China), CoronaVac (Sinovac, Beijing, China), Ad5-nCoV (CanSino Biologics, Tianjin, China), BBV152 (Bharat Biotech, Hyderabad, Telagana, India), EpiVacCorona (Vector Institute, Koltsovo, Novosibirsk Oblast, Russia) ZF2001 (Anhui Zhifei Longcom Biopharmaceutical Co. Ltd., Hefei City, Anhui Province, China), CoviVac (The Chumakov Centre at the Russian Academy of Sciences, Leninskiy Prospekt, Moscow, Russia). Some other vaccines developed by AstraZeneca and Novavax are under phase 3 clinical trials (https://www.cdc.gov/coronavirus/2019-ncov/vaccines/different-vaccines.html (accessed on Saturday, 17 April 2021)). As of 17 April 2021, more than 520 million doses of different vaccines have been administered to control COVID-19. In addition, other approaches such as plasma therapy, decoy-soluble ACE2 proteins, drug repurposing, natural products, and blocking peptides are underway. However, developing a new therapeutic agent is a time-consuming, highly challenging, and costly. Thus, there is a pressing need to identify a specific drug or vaccine against various SARS-CoV-2 proteins and develop them for the therapeutic of COVID-19.

In this study, we employed computational tools to screen a library of marine seaweed compounds against the Spike protein RBD of the SARS-CoV-2 UK variant (VUI 202012/01). It is anticipated that the outcome of this study may contribute to the existing knowledge of anti-COVID-19 compounds and thus may contribute to the development of effective drugs against the SARS-CoV-2 UK variant.

## 2. Results and Discussion

### 2.1. Homology Modeling and Validation of UK SARS-CoV-2 RBD

**Figure 1:**
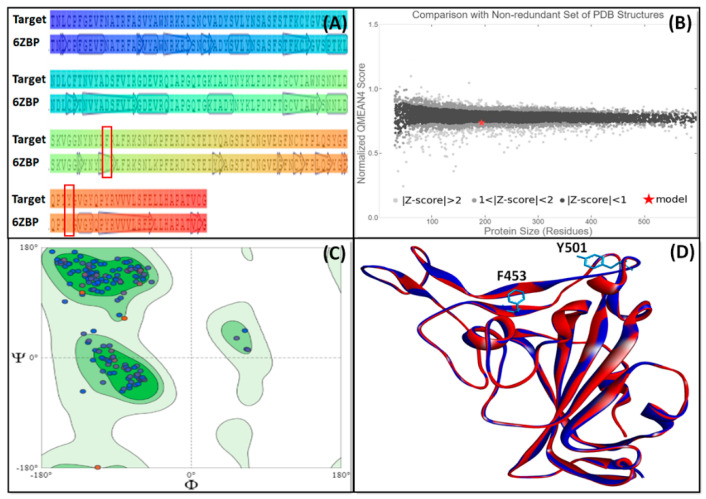
Homology modeling and validation of RBD from the Spike protein of UK SARS-CoV-2 strain. (**A**) Sequence alignment of target with the template (PDB ID: 6ZBP). The mutated residues (Y453F and N501Y) are highlighted in the red box, (**B**) QMEAN4 score of the generated model, as compared to the non-redundant set of PDB structures, (**C**) Ramachandran plot of the generated model, and (**D**) Superimposition of the generated model (blue color) on to the three-dimensional structure of the template (red color).

The three-dimensional model of the UK SARS-CoV-2 strain was generated by performing homology modeling in SWISS-MODEL. On the basis of sequence identity and sequence coverage, we considered chain A of 6ZBP as a template. The sequence alignment of UK SARS-CoV-2 RBD and chain A of 6ZBP is shown in Figure 1A, and the normalized QMEAN4 score of the generated model is presented in Figure 1B. The QMEAN4 score of the generated model was less than 1, which implies that its three-dimensional structure was comparable to the non-redundant set of PDB structures. Further, the overall quality of the generated RBD model was evaluated with the help of PSVS software to enumerate parameters such as Ramachandran, Verify3D, Procheck, and Molprobity clash scores (Table 1). Analysis of Ramachandran plot from Procheck and Richardson’s lab suggests that all the residues of the RBD model were with most favored and additional/generously allowed regions (Figure 1C and Table 1). It is noteworthy that none of the residues were in the disallowed regions. Moreover, RBD model and template superimposition suggested that RBD occupied a similar conformational space (Figure 1D). The relative positions of Tyr453Phe and Asn501Tyr mutations are also depicted.

### 2.2. HTVS, SP, and XP Molecular Docking of Marine Seaweed Compounds against RBD of Spike Protein

In structure-based drug design methodology, an extensive database comprising structurally diverse ligands is searched using HTVS and molecular docking to identify potential inhibitors [11,12]. In this study, we performed HTVS to screen compounds from a marine seaweed library against the RBD of SARS-CoV-2 Spike protein. In HTVS, 809 out of 1110 compounds present in the library (i.e., 72.9%) were able to bind RBD with binding energies varying from −6.951 to −0.333 kcal mol^−1^. The compounds with docking energy ≤−4.000 kcal mol^−1^ in HTVS (i.e., 51 compounds) were subjected to SP docking (Table S1). On the basis of SP docking, compounds with ≤−5.000 kcal mol^−1^ docking energy (i.e., five compounds) were further shortlisted and screened by XP docking. These compounds were BE011, GA004, GA005, GA006, and GA007; their structure, source, and chemical nature are presented in Table 2. The XP docking energy of these five compounds was between −5.439 and −8.326 kcal mol^−1^ (Table 2). On the basis of the lowest XP docking energy, we identified the compound BE011 (Dieckol) as the most potent binder of RBD and hence selected for further studies. Dieckol is abundantly isolated from the brown alga *Ecklonia cava* found on Jeju Island, Korea [13]. Dieckol has been reported to possess various biological properties such as anti-cancer, antithrombic and profibrinolytic activities, hepatoprotective, antioxidant, anti-diabetic, anti-hyperlipidemic, photochemopreventive, and anti-HIV [14,15,16,17,18,19,20,21,22,23]. Interestingly, Dieckol isolated from brown algae *Ecklonia cava* has been reported to possess inhibitory activity against 3CLpro or Mpro of SARS-CoV [24].

### 2.3. Investigation of Physicochemical Pharmacokinetic, Drug-Like, and Medicinal Properties

Determining physicochemical, pharmacokinetics, drug-like, and medicinal properties using in silico tools is widely accepted as a fast and accurate method [25]. In this study, the physicochemical and ADMET properties of the compound with lowest XP docking energy, i.e., BE011 or Dieckol, was determined. The results suggested that Dieckol was not suitable to be developed as a candidate drug molecule owing to the violations of Lipinski’s, Ghose’s Veber’s Egan’s, and Muegge’s rules (Table 3, Table 4 and Table 5). Lipinski’s rule of five suggests that an orally active drug should not have more than one violation of the following rules: molecular weight ≤ 500 Da, number of hydrogen bond donor ≤ 5, hydrogen bond acceptor ≤ 10, XlogP3 ≤ 5. We observed that Dieckol violated three Lipinski’s rules as it had a molecular weight of 742.55 Da, 11 hydrogen bond donors, and 18 hydrogen bond acceptors (Table 3). Moreover, Dieckol is not soluble and has poor gastrointestinal absorption. To improve the physicochemical and ADMET properties, we generated 10 derivatives of Dieckol (DK01-10) in silico using ChemSketch and optimized their energies using OPLS3e forcefield. The physicochemical and ADMET properties of all the Dieckol derivatives were determined using SwissADME, and the results are presented in Table 3, Table 4 and Table 5.

We observed that amongst all the Dieckol derivatives, DK07 (IUPAC name is 8-{3-hydroxy-4-[(7-hydroxynaphthalen-2-yl)oxy]phenoxy}-1,4-benzodioxin-5-ol) showed acceptable drug-like properties. The physicochemical properties of DK07, such as molecular weight, rotatable bonds, hydrogen bond donors, hydrogen bond acceptors, and XlogP3, were 416.38 Da, 4, 3, 9, and 4.88, respectively (Table 3). Most importantly, DK07 was moderately soluble and had a total polar surface area of 97.61 Å^2^. It displayed high gastrointestinal absorption and no blood–brain barrier crossing ability, and did not act as a P-glycoprotein substrate. DK07 acted as an inhibitor of CYP2C19, CYP2C9, CYP2D6, and CYP3A4. The skin permeability of DK07 was estimated to be −5.38 cm/s (Table 4). Most importantly, DK07 had only 1 Ghose’s violation and obeyed all the other drug-likeness filters such as Lipinski’s, Veber, Egan’s, and Muegge’s rules. It did not display any PAINS and Brenk alerts, although there were two lead-likeness alerts (Mol wt >350, and XLOGP3 >3.5). The bioavailability score and synthetic accessibility of DK07 were 0.55 and 3.70, respectively (Table 5). Since DK07 obeyed all the properties of a drug-like molecule and its ADMET properties were within acceptable limits, we analyzed the interaction between RBD and DK07 in detail and performed molecular dynamics and free energy calculation.

### 2.4. Interaction of Spike Protein RBD with ACE2 and Dieckol Derivative DK07

The RBD of SARS-CoV-2 Spike protein has emerged as the most suitable target for drug designing and development. It plays an essential role in recognizing ACE2 receptors on the host cell, thus mediating the internalization of viral RNA into the host. Here, we retrieved the X-ray crystal structure (PDB Id: 6M0J) of RBD in complex with ACE2 and analyzed the interaction pattern using BIOVIA Discovery Studio Visualizer [26]. We found that RBD and ACE2 interact through salt bridges, hydrogen bonds, and hydrophobic interactions (Table 6). Two salt bridges were formed by ACE2 Lys417:NZ with RBD Asp30:OD1 (3.09 Å) and Asp:OD2 (3.00 Å), while an electrostatic interaction was formed between Lys31:NZ of ACE2 and Glu484:OE1 of RBD (4.39 Å). Nine conventional hydrogen bonds were mediated by Gln24:OE1, Glu35:OE1, Asp38:OD2, Tyr41:OH, Gln42:NE2 (two bonds), Tyr83:OH, Lys353:NZ, and Lys353:O of ACE2 with Gly446:O, Tyr449:OH, Tyr449:OH, Asn487:OD1, Asn487:ND2, Gln493:NE2:B, Gly496:O, Thr500:OG1, and Gly502:N of RBD with a distance in the range of 2.69–3.24 Å (Table 6). We also observed one pi–donor hydrogen bond between ACE2 Tyr83:OH and RBD Phe486 (4.09 Å). The ACE2-RBD complex was further stabilized by six hydrophobic interactions between Lys31, His34, Met82, Tyr83, Lys353:C,O, and Gly354:N of ACE2 and Leu455:CD1, Phe486, Tyr489, and Tyr505 of RBD, with a distance in the range of 3.85–5.14 Å (Table 6). The previous report also suggested that a small stretch of 23 amino acid residues (Glu23 to Leu45) in the peptidase (PD) domain of ACE2 to be majorly involved in the interaction with RBD of Spike protein [27].

**Figure 2:**
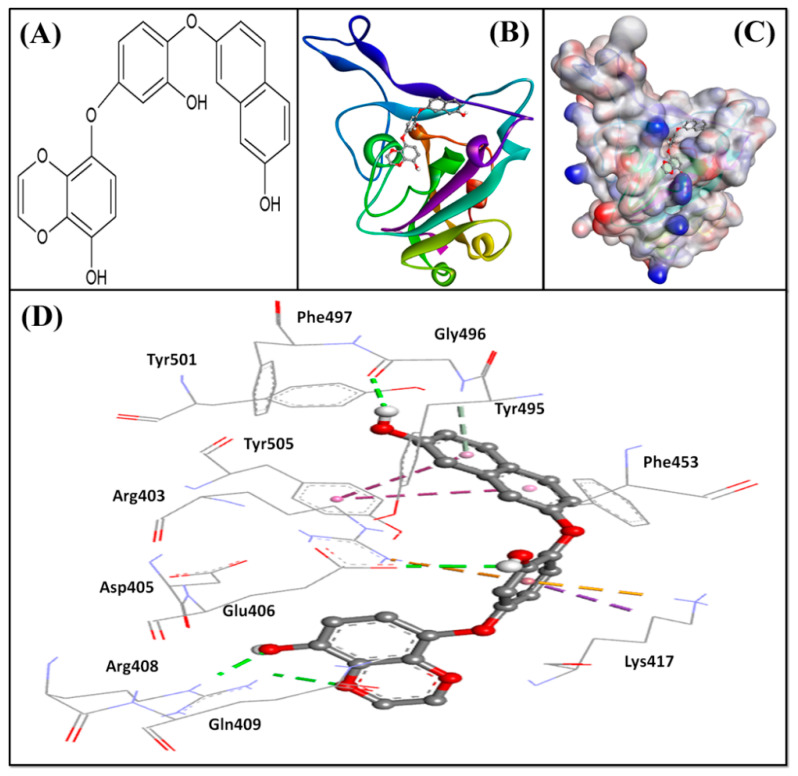
Molecular docking between DK07 and RBD of SARS-CoV-2 Spike protein in extra-precision (XP) mode. (**A**) 2D representation of Dieckol derivative DK07; (**A**,**B**) 2D representation showing binding of DK07 to the groove of RBD where ACE2 binds; (**C**) 3D representation showing binding of DK07 at the binding pocket of RBD; and (**D**) interaction between DK07 and RBD of Spike protein, showing the involvement of different amino acid residues and the molecular forces.

Further, we evaluated whether DK07 could bind to the interface of ACE2 and Spike protein RBD. Hence, extra precision (XP) molecular docking between DK07 and RBD (chain E of 6M0J) was performed using “Glide-2018” (Schrodinger, LLC, New York, NY, USA). An analysis of binding pose and interaction pattern between DK07 and Spike protein RBD revealed that DK07 was bound to the ACE2 binding groove of RBD through multiple interactions such as electrostatic interaction, hydrogen bonding, and hydrophobic interactions (Figure 2). The amino acid residues Arg403:NH2 and Lys417:HZ3 formed pi–cation electrostatic interactions with DK07. We observed four strong conventional hydrogen bonds formed by Glu406:OE2 (2.48 Å), Arg408:HE (2.11 Å), Arg408:HH21 (2.41 Å), and Gly496:O (2.43 Å), and three pi–donor hydrogen bonds formed by Arg403:NH2 (4.10 Å), Lys417:HZ3 (3.15 Å), and Gly496:HN (3.12 Å). Moreover, two pi–pi T-shaped hydrophobic interactions were formed by Tyr505 (5.92 and 5.17 Å), while Lys417:CD formed a pi–sigma hydrophobic interaction (3.83 Å). Other residues such as Asp405, Arg408, Gln409, Phe453, Tyr495, Phe497, Tyr501, and Tyr505 were engaged in Van der Waals’ interaction with DK07. Although Van der Waal’s force is usually weak, it plays significant role in enhancing the attraction and binding of ligands to protein. Interestingly, the amino acid residues Lys417, Gly496, and Tyr505 of Spike protein RBD were found to be commonly involved in the interaction with ACE2 and DK07, thus suggesting that DK07 was bound at the same location where ACE2 interacts with Spike protein RBD. The analysis of docking energy (-9.254 kcal mol^−1^) and the corresponding binding affinity (6.13 × 10^6^ M^−1^) of DK07 towards Spike protein RBD suggested a strong interaction between RBD and DK07 and hence strong inhibition potential of DK07 (Table 6).

### 2.5. MD (Molecular Dynamics) Simulation Analysis

MD simulation is a powerful tool to analyze the structure and dynamics of the protein–ligand complex. We performed 50 ns MD simulation of the RBD–DK07 complex and measured various parameters, as described below.

#### 2.5.1. RMSD (Root Mean Square Deviation) Calculations

**Figure 3:**
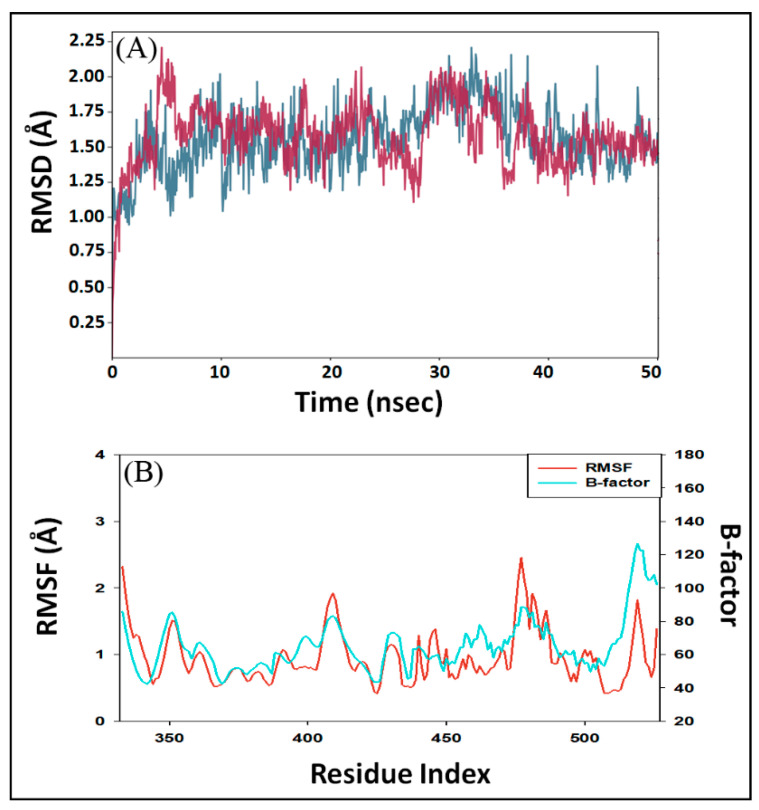
Molecular dynamics (MD) simulation of RBD–DK07 complex. (**A**) RMSD (root mean square deviation) of RBD alone (teal color) and in the presence of DK07 (brown color); (**B**) RMSF (root mean square fluctuation) of RBD in the presence of DK07 (red color), as compared with B-factor, which was determined during X-ray crystallography (teal color).

In MD simulation, RMSD is measured as a deviation in the structure of protein or protein–ligand complex with respect to a reference structure usually the initial frame. Figure 3A shows RMSD in Cα atoms of RBD alone (teal color) and RBD–DK07 complex (brown line). As compared to the initial frame, no significant fluctuations were observed in RMSD values of protein and protein–inhibitor complex throughout the simulation time. The mean RMSD values of RBD alone or in complex with DK07 were obtained as 1.5575 and 1.6305 Å, respectively. Since the variation in RMSD values of protein and protein–inhibitor complex were much lower than the acceptable limit of 2.0 Å, forming a stable RBD–DK07 complex was anticipated (Figure 3A).

#### 2.5.2. RMSF (Root Mean Square Fluctuation) Calculations

In MD simulation, RMSF value of a protein is generally measured to access the fluctuations in the side chains due to the binding of a ligand. Figure 3B depicts the RMSF of Spike protein RBD (teal color) in the presence of DK07 during MD simulation and compared it with the experimentally determined B-factor (red color) obtained during X-ray crystallography. Minor fluctuations in RMSF values of RBD side chains might have been due to the entry and binding of DK07 into the groove of the protein. Throughout the MD simulation, the RMSF values coincided with the B-factor values, thus suggesting that the binding of DK07 did not alter the overall conformation of RBD.

#### 2.5.3. Secondary Structure Analysis

**Figure 4:**
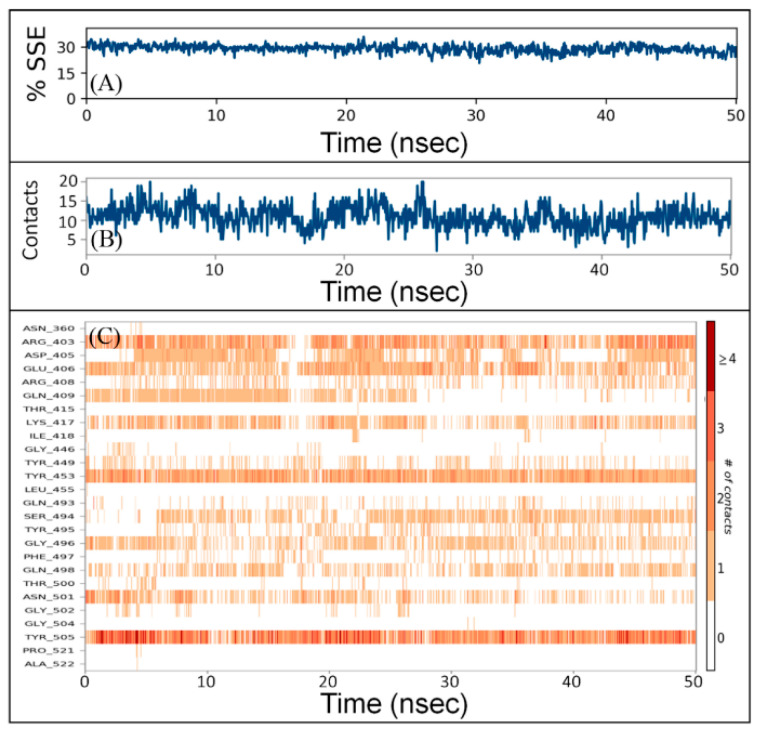
Interaction between RBD and DK07 during simulation. (**A**) Percentage of RBD–DK07 secondary structure element (SSE) varied during simulation, (**B**) the number of contacts between RBD and DK07 as a function of simulation, (**C**) participation of different amino acid residues of RBD in making contacts with DK07 as a function of simulation.

The interaction between a protein and inhibitor may alter the secondary structure elements (SSE) of the protein. In this study, we monitored the changes in SSE of RBD due to the binding of DK07 during simulation (Figure 4A). The total SSE of RBD in complex with DK07 was 29% (α-helix = 7% and β-sheets = 22%), which was in agreement with the reported values of SSEs 32% (α-helix = 11% and 21% β-sheets). The results indicated that the binding of DK07 to RBD did not considerably modify its secondary structure.

#### 2.5.4. Protein–Ligand Interaction Analysis

The total number of contacts between RBD and DK07 during simulation was determined to vary in the 4–20 range, with an average of 12 contacts (Figure 4B). Moreover, the involvement of amino acid residues in making contact with DK07 during simulation showed that Arg403, Glu406, Lys417, Tyr453, and Tyr505 were involved in making contact for most of the simulation (Figure 4C).

#### 2.5.5. Radius of Gyration (rGyr) and Different Surface Area Analysis

**Figure 5:**
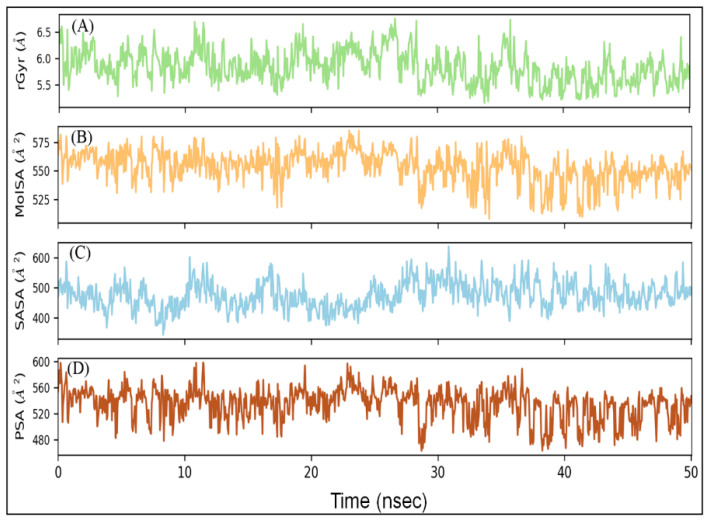
Variation in (**A**) rGyr (radius of gyration), (**B**) MSA (molecular surface area), (**C**) SASA (solvent accessible surface area), and (**D**) PSA (polar surface area) of RBD–DK07 complex during MD simulation.

The radius of gyration (rGyr) is considered a significant indicator of the protein’s folding state in different conditions. Here, the rGyr of the RBD–DK07 complex was measured to gain an insight into the compactness of protein during simulation (Figure 5A). The rGyr of RBD fluctuated between 5.16 and 6.75 Å, with an average value of 5.84 Å. The solvent-exposed surface area of a protein under different conditions is generally accessed to look for any conformational changes [28]. Here, molecular surface area (MolSA), solvent accessible surface area (SASA), and polar surface area (PSA) of RBD in complex with DK07 were measured during simulation to explore the exposure of the protein to the solvent molecules and thus to access its conformational stability (Figure 5B–D). MolSA, SASA, and PSA of DK07 were in the range of 508.36–585.17, 343.89–636.88, and 463.26–598.53 Å^2^, respectively. The average values of MSA, SASA, and PSA were estimated to be 553.62, 475.11, and 534.58 Å^2^, respectively. Although the values of surface areas fluctuated for the initial part of the simulation, they became stabilized and remained within acceptable error once favorable contacts were made between DK07 and RBD. The results of rGyr and surface areas confirmed the formation of a stable RBD–DK07 complex.

### 2.6. Free Energy (Prime/MM-GBSA) Calculation of RBD–DK07 Interaction

The binding of a ligand into the pocket of a protein is governed by the thermodynamic contribution of ligand to the overall binding free energy of the protein–ligand complex. Thus, the molecular interactions formed between ligand and protein play a significant role in determining the overall stability and affinity of a ligand inside the binding pocket of protein [27]. Therefore, we performed Prime/MM-GBSA to ascertain the binding thermodynamics of DK07 towards RBD. The binding free energy (Δ*G*_bind_) of DK07 towards RBD was estimated to be −52.87 kcal mol^−1^, which reflected a strong interaction between DK07 and RBD (Table 7). Although the Δ*G*_bind_ of the RBD–DK07 complex was significantly higher than the docking Δ*G* of RBD–DK07 interaction, it might have been due to the limitations of forcefield (OPLS3e) used in the analysis. Prime calculates MM-GBSA using the relative binding affinity of protein–ligand without considering any simulation process. Furthermore, previous reports suggest a disagreement between docking Δ*G* and MM-GBSA Δ*G*_bind_ determined using Prime module [29,30,31]. Many ligands have been shown to bind the RBD of Spike protein with an estimated value of Δ*G*_bind_ in the range of −21.45 to −54.11 kcal/mol, which is close to our estimated value [32,33]. Interestingly, the Prime/MM-GBSA values of lomitapide, dihydroergotamine mesylate, and olaparib against the Spike protein of SARS-CoV-2 have been estimated to be −98.46, −84.67, and −80.19, respectively [34]. The decomposition of binding free energy (Δ*G*_bind_) into its constituents revealed that gas phase energy (Δ*E*_gas_ = −127.75 kcal mol^−1^) favors the formation of the DK07–RBD complex, while solvation energy (Δ*G*_sol_ = 74.88 kcal mol^−1^) opposed it. Moreover, the polar (Δ*G*_polar_) and non-polar (Δ*G*_non-polar_) solvation energies, which comprised overall solvation energy (Δ*G*_sol_) of DK07–RBD interaction were found to be 78.67 and −3.79 kcal mol^−1,^ respectively. Further, the gas phase energy (Δ*E*_gas_) constituents such as Van der Waal’s energy (Δ*E*_vdW_ = −58.16 kcal mol^−1^) and electrostatic or Coulomb energy (Δ*E*_elec_ = −69.99 kcal mol^−1^) stabilized the DK07–RBD complex. These results were in agreement with the molecular docking results, suggesting that electrostatic interactions between DK07 and Arg403:NH1 and Glu406:OE2 along with Van der Waals’ interactions favored the binding of DK07 to RBD of Spike protein and stabilized the RBD–DK07 complex.

## 3. Materials and Methods

### 3.1. Preparation of Ligands

A library of marine seaweed metabolites containing 1110 unique compounds was retrieved from the Seaweed Metabolite Database [35]. The library harbors a diverse chemical space with known biological activities such as antiviral, antimicrobial, anti-inflammatory, and anti-cancer [36]. Before molecular docking, the conformation of ligands was optimized by removing salt (if any) and producing different ionization states at pH 7.0 ± 1.0 using “Epik module in LigPrep tool” (Schrodinger-2018-4, LLC, NY, USA) followed by energy minimization using the optimized potential for liquid simulation (OPLS3e) forcefield as described previously [26,37].

### 3.2. Homology Modeling of UK SARS-CoV-2 Spike Protein RBD and Its Validation

A model of UK SARS-CoV-2 Spike protein RBD was generated by inserting Tyr453Phe and Asn501Tyr mutations in the Spike protein RBD using PyMol. The primary amino acid sequence of the mutated strain was used to search the templates in SWISS-MODEL. On the basis of GMQE score and percent identity, we used chain A of 6ZBP as a template to generate the model using SWISS-MODEL. The three-dimensional structure of the generated model was validated using the protein structure validation suite (PSVS). Parameters such as PROCHECK, VERIFY3D, and Ramachandran plot were generated and examined. Further, ProSA-web was used to assess the overall quality of the model.

### 3.3. Preparation of Target Protein (Spike Protein RBD of UK SARS-CoV-2)

The 3D coordinates of Spike protein RBD of SARS-CoV-2 (PDB Id: 6M0J; resolution 2.45 Å) were retrieved from the PDB-RCSB database [38]. In the X-ray crystal structure, Spike protein RBD was in complex with the ACE2 receptor protein. Before molecular docking, Spike protein RBD was pre-processed, deleting any heterogeneous atoms or ligands including ACE2 and removing non-catalytic water molecules. The bond orders were defined, and missing H-atoms were added using “Protein Preparation Wizard” (Schrodinger-2018-4, LLC, New York, NY, USA), as reported previously [37,39]. A network of H-bonds was defined, and the whole system was energy minimized through the OPLS3e forcefield. The ligand conformational search for molecular docking was accomplished inside a grid box of 80 × 80 × 80 Å dimension (located at −37.0 × 29.8 × 4.4 Å) generated by the “Receptor-Grid Generation Tool” (Schrodinger-2018-4, LLC, New York, NY, USA).

### 3.4. Molecular Docking

The library of marine seaweed compounds was screened against the Spike protein RBD at 3 different stages, namely, high-throughput virtual screening (HTVS), standard precision (SP) molecular docking, and extra precision (XP) molecular docking in “Glide” (Schrodinger-2018-4, LLC, New York, NY, USA), as reported previously [40,41]. The top-scoring compounds with a docking score ≤ −4.000 kcal mol^−1^ in HTVS (51 ligands) were subjected to SP docking. The most promising compounds with a docking score ≤ −5.000 kcal mol^−1^ in SP docking (5 ligands) were subjected to XP docking. The binding affinity (*K*_d_) of the ligands was calculated from the docking energy (Δ*G*) for Spike protein RBD using the below equation [42,43].

Δ*G* = −*RT* ln K_d_ (1)

where *R* and *T* are Boltzmann gas constant (=1.987 cal/mol/K) and temperature (=298 K), respectively.

### 3.5. Physicochemical, Drug-Like, and ADMET Properties

The physicochemical, pharmaceutical, medicinal, drug-like, and ADMET (absorption, distribution, metabolism, excretion, and toxicity) properties of ligand(s) were determined using SwissADME [44,45,46].

### 3.6. Molecular Dynamics (MD) Simulation

The dynamics and stability of protein–ligand complex were evaluated by performing MD simulation with the help of “Desmond” (Schrodinger-2018-4, LLC, New York, NY, USA). An orthorhombic simulation box was used to perform MD simulation after placing the protein–ligand complex at the center of the box, at least 10 Å away from the boundaries. TIP3P explicit solvent molecules were used to solvate the simulation box, and the system was neutralized by adding proper counterions. The physiological conditions were mimicked by adding 150 mM NaCl and adjusting the system’s temperature and pressure to 298 K and 1 atm bar. The energy of system was minimized by 2000 iterations with convergence criteria of 1 kcal/mol/Å using OPLS3e forcefield. A production run of 50 ns was computed using NTP (isobaric-isothermal) ensemble. The temperature was maintained using a Nose-Hoover Chain thermostat, and the pressure was kept constant with the help of Matrtyna–Tobias–Klein barostat [47,48]. During the production run, a time step of 2 fs was fixed, and the energy and structure were logged at an interval of 10 ps in the trajectory. Analysis of MDS trajectory was performed using “Simulation Interaction Diagram tool” (Schrodinger, LLC, NY, USA) to determine the root mean square deviation (RMSD), root mean square fluctuation (RMSF), radius of gyration (rGyr), molecular surface area (MolSA), solvent accessible surface area (SASA), and polar surface area (PSA).

### 3.7. Determination of Binding Free Energy Using Prime/MM-GBSA

The binding free energy of ligand having lowest XP docking energy was determined using the molecular mechanics (MM) and generalized Born surface area (GBSA) using “Prime/MM-GBSA module” (Schrodinger-2018-4, LLC, New York, NY, USA) as reported previously [49]. The binding free energy (Δ*G*_bind_) of the protein–ligand complex was measured using the following relations:

Δ*G*_bind_ = *G*(complex) − [*G*(protein) + *G*(ligand)] (2)

Δ*G*_bind_ = Δ*H* − *T*Δ*S* (3)

Δ*H* = Δ*E*_gas_ + Δ*G*_sol_ (4)

Δ*E*_gas_ = *E*_int_ + *E*_vdW_ + *E*_elec_ (5)

*E*_int_ = *E*_bond_ + *E*_angle_ + *E*_torsion_ (6)

*G*_sol_ = *G*_polar_ + *G*_non-polar_ (7)

*G*_non-polar_ = γSASA + β (8)

The binding free energy (Δ*G*_bind_) is the difference in free energy of the complex and the individual free energies of protein and ligand. In other words, binding free energy is defined as the difference between enthalpic (Δ*H*) and entropic contributions (*T*Δ*S*), where enthalpy (Δ*H*) is the sum of gas phase energy (Δ*E*_gas_) and solvation free energy (Δ*G*_sol_). Further, the gas phase energy (Δ*E*_gas_) is the sum of internal (*E*_int_), Van der Waals’ (*E*_vdW_), and electrostatic or Coulombic (*E*_elec_) energies. The internal energy (*E*_int_) term can further be divided into energies of the bonds (*E*_bond_), angles (*E*_angle_), and torsions (*E*_torsion_). Conversely, solvation free energy (*G*_sol_) is the total of polar (*G*_polar_) and non-polar (*G*_non-polar_) solvation energies. The polar solvation energy (*G*_polar_) was determined by employing the generalized Born (GB) implicit solvation model. The non-polar solvation energy was calculated by taking the values of surface tension proportionality constant (γ) as 0.00542 kcal mol^−1^ Å^2^, and non-polar solvation free energy of a point solute (β) as 0 kcal mol^−1^. Moreover, SASA was calculated using a linear combination of pairwise overlap (LCPO) model.

## 4. Conclusions

Computational tools have been found to reduce time and effort to identify a potential therapeutic solution against different diseases [50,51,52]. The present study focused on identifying potential compounds that can block ACE2-RBD interaction. We screened a library of marine seaweed compounds against the UK strain of SARS-CoV-2 harboring multiple mutations in Spike protein. We employed different computational tools such as homology modeling, HTVS, molecular docking, molecular dynamic simulation, and free energy calculations. In view of the absence of three-dimensional crystal structure of RBD of UK SARS-CoV-2 strain, we generated a homology model of RBD using SWISS-MODEL. On the basis of extra precision molecular docking scores, we identified BE011 (Dieckol) as the most promising inhibitor of the RBD model. Dieckol is abundantly found in brown alga *Ecklonia cava* from Jeju Island, Korea. The structure analysis of Dieckol suggests that it does not possess drug-like properties and hence cannot be used as a lead inhibitor against RBD. Further, we utilized the structure of Dieckol as scaffold to generate different derivatives using ChemSketch and subjected them to XP molecular docking and ADMET analysis. The Dieckol derivative, i.e., DK07 was identified as the most potent inhibitor of RBD and was found to possess acceptable physicochemical, pharmacokinetic, drug-likeness, and ADMET properties. The IUPAC name of DK07 is 8-{3-hydroxy-4-[(7-hydroxynaphthalen-2-yl)oxy]phenoxy}-1,4-benzodioxin-5-ol. DK07 binds to the RBD at the ACE2–RBD interface and interacts with key amino acid residues. Free energy calculation and molecular dynamics simulation of RBD–DK07 suggest the involvement of hydrogen bonding, electrostatic interactions, and Van der Waals’ interactions as the driving source in the formation of a stable RBD–DK07 complex. The findings of this study require additional proof from in vitro and in vivo experiments to validate the potential of DK07 to bind UK strain of SARS-CoV-2 Spike protein RBD and prevent its interaction with ACE2.

## Figures and Tables

**Table 1:** Structural assessment of UK SARS-CoV-2 RBD model using Protein Structure Validation Suite (PSVS) software.

Parameters	Mean Score	Z-Score
Procheck G-factor (phi/psi only)	−0.34	−1.02
Procheck G-factor (all dihedral angles)	−0.16	−0.95
Verify 3D	0.21	−4.01
ProsaII (-ve)	0.45	−0.83
Procheck (*ϕ − ψ*)	−0.34	−1.02
Procheck (all)	−0.16	−0.95
Molprobity Clash score	1.34	1.30
RMSD_bond length (Å)	0.016	-
RMSD_bond angle (^o^)	1.9	-
Close contacts (within 2.2 Å)	0	-
Ramachandran plot summary (Procheck)		
Most favored regions (%) Additionally allowed regions (%) Generously allowed regions (%) Disallowed regions	90.4 9.6 0.0 0.0	-
Ramachandran plot statistics (Richardson’s lab)		
Most favored regions (%) Allowed regions (%) Disallowed regions (%)	97.9 2.1 0.0	-

**Table 2:** Extra-precision (XP) molecular docking of selected ligands (having ≤−5.000 kcal mol−1 in SP docking) against RBD of spike protein.

S. No.	Compound ID	Source	Structure and Chemical Name	Docking Score (kcal mol^−1^)	Binding Affinity (M^−1^)
1.	BE011 (Dieckol)	*Ecklonia cava*	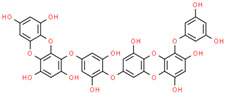 4-(4-{[6-(3,5-Dihydroxyphenoxy)-4,7,9-trihydroxyoxanthren-2-yl]oxy}-3,5-dihydroxyphenoxy)oxanthrene-1,3,6,8-tetrol	−8.326	1.28 × 10^6^
2.	GA004 (Nigricanoside A)	*Avrainvillea nigricans*	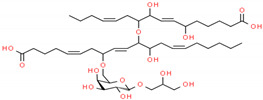 2,3-Dihydroxypropyl(5ξ)-6-*O*-[(4Z,8E,13Z)-1-carboxy-10-{[(4Z,9E)-15-carboxy-8,11-dihydroxy-4,9-pentadecadien-7-yl]oxy}-11-hydroxy-4,8,13-nonadecatrien-7-yl]-α-L-arabino-hexopyranoside	−7.952	6.80 × 10^5^
3.	GA005 (Nigricanoside B)	*Avrainvillea nigricans*	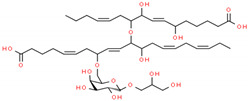 2,3-Dihydroxypropyl-(5ξ)-6-*O*-[(4*Z*,8*E*,13*Z*,16*Z*)-1-carboxy-10-{[(4*Z*,9*E*)-15-carboxy-8,11-dihydroxy-4,9-pentadecadien-7-yl]oxy}-11-hydroxy-4,8,13,16-nonadecatetraen-7-yl]-α-L-arabino-hexopyranoside	−6.962	1.28 × 10^5^
4.	GA006 (Nigricanoside A dimethyl ester)	*Avrainvillea nigricans*	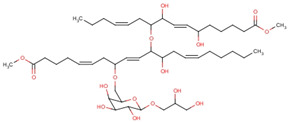 2,3-Dihydroxypropyl-6-*O*-[(5*Z*,9*E*,14*Z*)-11-{[(4*Z*,9*E*)-8,11-dihydroxy-16-methoxy-16-oxo-4,9-hexadecadien-7-yl]oxy}-12-hydroxy-1-methoxy-1-oxo-5,9,14-icosatrien-8-yl]-β-d-galactopyranoside	−5.703	1.52 × 10^4^
5.	GA007 (Nigricanoside B dimethyl ester)	*Avrainvillea nigricans*	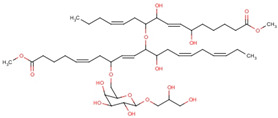 Methyl-(5*Z*,9*E*,14*Z*,17*Z*)-11-{[(4*Z*,9*E*)-8,11-dihydroxy-16-methoxy- 16-oxohexadeca-4,9-dien-7-yl]oxy}-8-{[6-(2,3-dihydroxy propoxy)-3,4,5-trihydroxyoxan-2-yl] methoxy}-12-hydroxyicosa-5,9,14,17-tetraenoate	−5.439	9.76 × 10^3^

**Table 3:** Physicochemical properties of Dieckol and its derivatives using SwissADME.

Name of Compound	MW (Da)	RB	HBA	HBD	TPSA (Å^2^)	Lipophilicity (XlogP3)	Solubility (logS)
Dieckol	742.55	6	18	11	287.14	4.87	Poor
DK01	538.46	4	10	4	136.3	5.83	Poor
DK02	528.59	4	5	4	90.15	7.79	Poor
DK03	558.62	4	6	4	99.38	8.06	Poor
DK04	550.51	4	9	5	138.07	5.5	Poor
DK05	578.56	4	9	7	160.07	5.58	Poor
DK06	472.4	4	9	3	116.07	4.77	Poor
DK07	416.38	4	7	3	97.61	4.88	Moderate
DK08	426.42	4	6	4	99.38	6.06	Poor
DK09	410.42	4	5	3	79.15	6.41	Poor
DK10	414.41	4	6	3	88.38	5.5	Poor

MW: molecular weight; RB: rotatable bonds; HBA: H-bond acceptor; HBD: H-bond donor; TPSA: total polar surface area.

**Table 4:** Pharmacokinetic properties of Dieckol and its derivatives using SwissADME.

Name of Compound	GIA	BBB	P-gp Substrate	Inhibitor of					log*K*_p_ (cm/s)
				CYP1A2	CYP2C19	CYP2C9	CYP2D6	CYP3A4	
Dieckol	Low	No	No	No	No	Yes	No	No	−7.37
DK01	Low	No	No	No	No	No	No	No	−5.45
DK02	Low	No	No	Yes	Yes	No	No	No	−3.99
DK03	Low	No	No	No	No	No	No	No	−3.98
DK04	Low	No	No	No	No	No	No	No	−5.75
DK05	Low	No	No	No	No	No	No	No	−5.87
DK06	Low	No	No	No	No	Yes	No	Yes	−5.79
DK07	High	No	No	No	Yes	Yes	Yes	Yes	−5.38
DK08	Low	No	No	No	Yes	Yes	Yes	No	−4.6
DK09	Low	No	Yes	No	Yes	Yes	Yes	No	−4.25
DK10	High	No	No	Yes	Yes	Yes	Yes	No	−4.92

GIA: gastrointestinal absorption; BBB: blood–brain barrier; P-gp: P-glycoprotein; log*K*_p_: skin permeability.

**Table 5:** Drug-like and medicinal properties of Dieckol and its derivatives using SwissADME.

Name of Compound	Number of Violations in					Bioavailability Score	PAINS	Brenk Alert	Lead-likeness Alert *	Synthetic Accessibility
	Lipinski	Ghose	Veber	Egan	Muegge		Alert			
Dieckol	3	4	1	2	5	0.55	0	0	2	4.68
DK01	1	3	0	2	1	0.55	0	0	2	3.97
DK02	2	3	0	1	1	0.17	0	0	2	4.07
DK03	2	4	0	1	1	0.17	0	0	2	4.93
DK04	1	3	0	2	1	0.55	0	0	2	4.59
DK05	2	3	1	1	3	0.17	0	0	2	4.6
DK06	0	1	0	1	0	0.55	0	0	2	4.05
DK07	0	1	0	0	0	0.55	0	0	2	3.7
DK08	0	1	0	1	1	0.55	0	0	2	2.92
DK09	0	1	0	1	1	0.55	0	0	2	2.88
DK10	0	1	0	0	1	0.55	0	0	2	3.78

* Mol wt > 350; XLOGP3 > 3.5.

**Table 6:** Molecular interaction of ACE2 and Dieckol derivative DK07 with RBD.

Donor-Acceptor Atoms *	Bond Distance (Å)	Type of Interaction	Δ*G*	*K*_d_
			(kcal mol^−1^)	(M^−1^)
***ACE2***				
E:Lys417:NZ—A:Asp30:OD2	3	Salt bridge	n.d.	n.d.
E:Lys417:NZ—A:As30:OD1	3.9	Salt bridge		
A:Lys31:NZ—E:Glu484:OE1	4.39	Electrostatic interaction		
A:Tyr41:OH—E:Thr500:OG1	2.7	Conventional hydrogen bond		
A:Gln42:NE2—E:Gly446:O	3.24	Conventional hydrogen bond		
A:Gln42:NE2—E:Tyr449:OH	2.78	Conventional hydrogen bond		
A:Tyr83:OH—E:Asn487:OD1	2.78	Conventional hydrogen bond		
A:Lys353:NZ—E:Gly496:O	3.08	Conventional hydrogen bond		
E:Tyr449:OH—A:Asp38:OD2	2.69	Conventional hydrogen bond		
E:Asn487:ND2—A:Gln24:OE1	2.69	Conventional hydrogen bond		
E:Gln493:NE2:B—A:Glu35:OE1	3.13	Conventional hydrogen bond		
E:Gly502:N—A:Lys353:O	2.78	Conventional hydrogen bond		
A:His34:CD2—E:Tyr453:OH	2.86	Carbon hydrogen bond		
A:Tyr83:OH—E:Phe486	4.09	Pi-donor hydrogen bond		
E:Leu455:CD1—A:His34	3.85	Hydrophobic (pi–sigma)		
A:Tyr83—E:Phe486	5.14	Hydrophobic (pi–pi stacked)		
A:Lys353:C,O;Gly354:N—E:Tyr505	4	Hydrophobic (amide–pi stacked)		
E:Phe486—A:Met82	4.74	Hydrophobic (pi–alkyl)		
E:Tyr489—A:Lys31	4.75	Hydrophobic (pi–alkyl)		
E:Tyr505—A:Lys353	4.62	Hydrophobic (pi–alkyl)		
*DK07 (8-{3-hydroxy-4-[(7-hydroxynaphthalen-2-yl)oxy]phenoxy}-1,4-benzodioxin-5-ol)*				
Arg408:HE—UNK:O	2.11	Conventional hydrogen bond	−8.954	3.69 × 10^6^
Arg408:HH21—UNK:O	2.41	Conventional hydrogen bond		
UNK:H—Glu406:OE2	2.48	Conventional hydrogen bond		
UNK:H—Gly496:O	2.43	Conventional hydrogen bond		
Arg403:NH2—UNK	4.1	Pi–cation; pi–donor hydrogen bond		
Lys417:HZ3—UNK	3.15	Pi–cation; pi–donor hydrogen bond		
Gly496:HN—UNK	3.12	Pi–donor hydrogen bond		
Lys417:CD—UNK	3.83	Hydrophobic (pi–sigma)		
Tyr505—UNK	5.92	Hydrophobic (pi–pi T-shaped)		
Tyr505—UNK	5.17	Hydrophobic (pi–pi T-shaped)		

* The polypeptide chains A and E belong to ACE2 and RBD of SARS-CoV-2 Spike protein; ΔG is docking energy; *K*_d_ is binding affinity; n.d. stands for not determined.

**Table 7:** Prime/MM-GBSA energies of Spike protein RBD–DK07 complex.

Binding Free Energy (Δ*G*_bind_)	Solvation Energy (Δ*G*_sol_)	Gas Phase Energy (Δ*E*_gas_)	Coulomb Energy (Δ*E*_elec_)	Van der Waals Energy (Δ*E*_vdW_)	Internal Energy (Δ*E*_int_)	Polar Solvation Energy (Δ*G*_polar_)	Non-Polar Solvation Energy (Δ*G*_non-polar_)
−52.87	74.88	−127.75	−69.99	−58.16	0.40	78.67	−3.79

All the energies are in kcal mol^−1^.

## Supplementary Materials

The following are available online at https://www.mdpi.com/article/10.3390/md19050242/s1. Table S1: Standard-precision (SP) molecular docking of selected ligands (having ≤−4.000 kcal mol^−1^ in HTVS) against RBD of the spike protein. Figure S1: Two-dimensional structures of Dieckol and its derivatives (DK01-DK10) used in the study.

## References

[1] Cucinotta D., Vanelli M. (2020). WHO declares COVID-19 a pandemic. Acta Biomed..

[2] Guo Y.R., Cao Q.D., Hong Z.S., Tan Y.Y., Chen S.D., Jin H.J., Tan K.S., Wang D.Y., Yan Y. (2020). The origin, transmission and clinical therapies on coronavirus disease 2019 (COVID-19) outbreak- A n update on the status. Mil. Med. Res..

[3] She J., Jiang J., Ye L., Hu L., Bai C., Song Y. (2020). 2019 novel coronavirus of pneumonia in Wuhan, China: Emerging attack and management strategies. Clin. Transl. Med..

[4] Song Z., Xu Y., Bao L., Zhang L., Yu P., Qu Y., Zhu H., Zhao W., Han Y., Qin C. (2019). From SARS to MERS, Thrusting Coronaviruses into the Spotlight. Viruses.

[5] Chen Y., Liu Q., Guo D. (2020). Emerging coronaviruses: Genome structure, replication, and pathogenesis. J. Med. Virol..

[6] Oude Munnink B.B., Nieuwenhuijse D.F., Stein M., O’Toole Á., Haverkate M., Mollers M., Kamga S.K., Schapendonk C., Pronk M., Lexmond P. (2020). Rapid SARS-CoV-2 whole-genome sequencing and analysis for informed public health decision-making in the Netherlands. Nat. Med..

[7] Lai C.C., Shih T.P., Ko W.C., Tang H.J., Hsueh P.R. (2020). Severe acute respiratory syndrome coronavirus 2 (SARS-CoV-2) and coronavirus disease-2019 (COVID-19): The epidemic and the challenges. Int. J. Antimicrob. Agents.

[8] Palese L.L. (2020). The Structural Landscape of SARS-CoV-2 Main Protease: Hints for Inhibitor Search. ChemRxiv.

[9] Yang H., Bartlam M., Rao Z. (2006). Drug Design Targeting the Main Protease, the Achilles Heel of Coronaviruses. Curr. Pharm. Des..

[10] Lu R., Zhao X., Li J., Niu P., Yang B., Wu H., Wang W., Song H., Huang B., Zhu N. (2020). Genomic characterisation and epidemiology of 2019 novel coronavirus: Implications for virus origins and receptor binding. Lancet.

[11] Kuntz I.D., Blaney J.M., Oatley S.J., Langridge R., Ferrin T.E. (1982). A geometric approach to macromolecule-ligand interactions. J. Mol. Biol..

[12] Faheem M., Rehman M.T., Danishuddin M., Khan A.U. (2013). Biochemical Characterization of CTX-M-15 from Enterobacter cloacae and Designing a Novel Non-β-Lactam-β-Lactamase Inhibitor. PLoS ONE.

[13] Li Y., Qian Z.J., Ryu B.M., Lee S.H., Kim M.M., Kim S.K. (2009). Chemical components and its antioxidant properties in vitro: An edible marine brown alga, Ecklonia cava. Bioorganic Med. Chem..

[14] Ahn M.J., Yoon K.D., Min S.Y., Lee J.S., Kim J.H., Kim T.G., Kim S.H., Kim N.G., Huh H., Kim J. (2004). Inhibition of HIV-1 reverse transcriptase and protease by phlorotannins from the brown alga Ecklonia cava. Biol. Pharm. Bull..

[15] Hwang H., Chen T., Nines R.G., Shin H.C., Stoner G.D. (2006). Photochemoprevention of UVB-induced skin carcinogenesis in SKH-1 mice by brown algae polyphenols. Int. J. Cancer.

[16] Yoon N.Y., Kim H.R., Chung H.Y., Choi J.S. (2008). Anti-hyperlipidemic effect of an edible brown algae, Ecklonia stolonifera, and its constituents on poloxamer 407-induced hyperlipidemic and cholesterol-fed rats. Arch. Pharmacal Res..

[17] Heo S.J., Ko S.C., Cha S.H., Kang D.H., Park H.S., Choi Y.U., Kim D., Jung W.K., Jeon Y.J. (2009). Effect of phlorotannins isolated from Ecklonia cava on melanogenesis and their protective effect against photo-oxidative stress induced by UV-B radiation. Toxicol. Vitr..

[18] Lee S.H., Park M.H., Heo S.J., Kang S.M., Ko S.C., Han J.S., Jeon Y.J. (2010). Dieckol isolated from Ecklonia cava inhibits α-glucosidase and α-amylase in vitro and alleviates postprandial hyperglycemia in streptozotocin-induced diabetic mice. Food Chem. Toxicol..

[19] Moon H.E., Islam M.N., Ahn B.R., Chowdhury S.S., Sohn H.S., Jung H.A., Choi J.S. (2011). Protein tyrosine phosphatase 1B and α-glucosidase inhibitory phlorotannins from edible brown algae, Ecklonia stolonifera and Eisenia bicyclis. Biosci. Biotechnol. Biochem..

[20] Kang M.C., Ahn G., Yang X., Kim K.N., Kang S.M., Lee S.H., Ko S.C., Ko J.Y., Kim D., Kim Y.T. (2012). Hepatoprotective effects of dieckol-rich phlorotannins from Ecklonia cava, a brown seaweed, against ethanol induced liver damage in BALB/c mice. Food Chem. Toxicol..

[21] Kim T.H., Ku S.K., Bae J.S. (2012). Antithrombotic and profibrinolytic activities of eckol and dieckol. J. Cell. Biochem..

[22] Wang C.H., Li X.F., Jin L.F., Zhao Y., Zhu G.J., Shen W.Z. (2019). Dieckol inhibits non-small–cell lung cancer cell proliferation and migration by regulating the PI3K/AKT signaling pathway. J. Biochem. Mol. Toxicol..

[23] Park S.J., Kim Y.T., Jeon Y.J. (2012). Antioxidant dieckol downregulates the Rac1/ROS signaling pathway and inhibits Wiskott-Aldrich syndrome protein (WASP)-family verprolin-homologous protein 2 (WAVE2)-mediated invasive migration of B16 mouse melanoma cells. Mol. Cells.

[24] Park J.Y., Kim J.H., Kwon J.M., Kwon H.J., Jeong H.J., Kim Y.M., Kim D., Lee W.S., Ryu Y.B. (2013). Dieckol, a SARS-CoV 3CLpro inhibitor, isolated from the edible brown algae Ecklonia cava. Bioorganic Med. Chem..

[25] Kumar A., Rathi E., Kini S.G. (2019). E-pharmacophore modelling, virtual screening, molecular dynamics simulations and in-silico ADME analysis for identification of potential E6 inhibitors against cervical cancer. J. Mol. Struct..

[26] Sastry G.M., Adzhigirey M., Day T., Annabhimoju R., Sherman W. (2013). Protein and ligand preparation: Parameters, protocols, and influence on virtual screening enrichments. J. Comput. Aided. Mol. Des..

[27] Baig M.S., Alagumuthu M., Rajpoot S., Saqib U. (2020). Identification of a Potential Peptide Inhibitor of SARS-CoV-2 Targeting its Entry into the Host Cells. Drugs R D.

[28] Rodier F., Bahadur R.P., Chakrabarti P., Janin J. (2005). Hydration of protein-protein interfaces. Proteins Struct. Funct. Genet..

[29] Gupta P., Khan S., Fakhar Z., Hussain A., Rehman M.T., Alajmi M.F., Islam A., Ahmad F., Hassan M.I. (2020). Identification of Potential Inhibitors of Calcium/Calmodulin-Dependent Protein Kinase IV from Bioactive Phytoconstituents. Oxidative Med. Cell. Longev..

[30] AlAjmi M.F., Rehman M.T., Hussain A., Rather G.M. (2018). Pharmacoinformatics approach for the identification of Polo-like kinase-1 inhibitors from natural sources as anti-cancer agents. Int. J. Biol. Macromol..

[31] Rehman M.T., Alajmi M.F., Hussain A., Rather G.M., Khan M.A. (2019). High-throughput virtual screening, molecular dynamics simulation, and enzyme kinetics identified ZINC84525623 as a potential inhibitor of NDM-1. Int. J. Mol. Sci..

[32] Unni S., Aouti S., Thiyagarajan S., Padmanabhan B. (2020). Identification of a repurposed drug as an inhibitor of Spike protein of human coronavirus SARS-CoV-2 by computational methods. J. Biosci..

[33] Sethi A., Sanam S., Munagalasetty S., Jayanthi S., Alvala M. (2020). Understanding the role of galectin inhibitors as potential candidates for SARS-CoV-2 spike protein:: In silico studies. RSC Adv..

[34] De Vita S., Chini M.G., Lauro G., Bifulco G. (2020). Accelerating the repurposing of FDA-approved drugs against coronavirus disease-19 (COVID-19). RSC Adv..

[35] Dicky G., Davis J., Hannah A., Vasanthi R. (2011). Bioinformation Seaweed Metabolite Database (SWMD): A Database of Natural Compounds from Marine Algae.

[36] Gallimore W. (2017). Marine Metabolites: Oceans of Opportunity. Pharmacognosy: Fundamentals, Applications and Strategy.

[37] Liang J., Pitsillou E., Karagiannis C., Darmawan K.K., Ng K., Hung A., Karagiannis T.C. (2020). Interaction of the prototypical α-ketoamide inhibitor with the SARS-CoV-2 main protease active site in silico: Molecular dynamic simulations highlight the stability of the ligand-protein complex. Comput. Biol. Chem..

[38] Lan J., Ge J., Yu J., Shan S., Zhou H., Fan S., Zhang Q., Shi X., Wang Q., Zhang L. (2020). Structure of the SARS-CoV-2 spike receptor-binding domain bound to the ACE2 receptor. Nature.

[39] Halgren T.A. (2009). Identifying and characterizing binding sites and assessing druggability. J. Chem. Inf. Model..

[40] Halgren T.A., Murphy R.B., Friesner R.A., Beard H.S., Frye L.L., Pollard W.T., Banks J.L. (2004). Glide: A New Approach for Rapid, Accurate Docking and Scoring. 2. Enrichment Factors in Database Screening. J. Med. Chem..

[41] Friesner R.A., Murphy R.B., Repasky M.P., Frye L.L., Greenwood J.R., Halgren T.A., Sanschagrin P.C., Mainz D.T. (2006). Extra precision glide: Docking and scoring incorporating a model of hydrophobic enclosure for protein-ligand complexes. J. Med. Chem..

[42] Rehman M.T., Ahmed S., Khan A.U. (2016). Interaction of meropenem with ‘N’ and ‘B’ isoforms of human serum albumin: A spectroscopic and molecular docking study. J. Biomol. Struct. Dyn..

[43] Rehman M.T., Shamsi H., Khan A.U. (2014). Insight into the Binding Mechanism of Imipenem to Human Serum Albumin by Spectroscopic and Computational Approaches. Mol. Pharm..

[44] Jabir N.R., Shakil S., Tabrez S., Khan M.S., Rehman M.T., Ahmed B.A. (2020). In silico screening of glycogen synthase kinase-3β targeted ligands against acetylcholinesterase and its probable relevance to Alzheimer’s disease. J. Biomol. Struct. Dyn..

[45] Sun H., Li Y., Tian S., Xu L., Hou T. (2014). Assessing the performance of MM/PBSA and MM/GBSA methods. 4. Accuracies of MM/PBSA and MM/GBSA methodologies evaluated by various simulation protocols using PDBbind data set. Phys. Chem. Chem. Phys..

[46] Daina A., Zoete V. (2016). A BOILED-Egg To Predict Gastrointestinal Absorption and Brain Penetration of Small Molecules. ChemMedChem.

[47] Brańka A.C. (2000). Nosé-Hoover chain method for nonequilibrium molecular dynamics simulation. Phys. Rev. E.

[48] Martyna G.J., Tobias D.J., Klein M.L. (1994). Constant pressure molecular dynamics algorithms. J. Chem. Phys..

[49] Genheden S., Ryde U. (2015). The MM/PBSA and MM/GBSA methods to estimate ligand-binding affinities. Expert Opin. Drug Discov..

[50] Ton A.T., Gentile F., Hsing M., Ban F., Cherkasov A. (2020). Rapid Identification of Potential Inhibitors of SARS-CoV-2 Main Protease by Deep Docking of 1.3 Billion Compounds. Mol. Inform..

[51] Mittal L., Kumari A., Srivastava M., Singh M., Asthana S. (2020). Identification of potential molecules against COVID-19 main protease through structure-guided virtual screening approach. J. Biomol. Struct. Dyn..

[52] Kumar N., Srivastava R., Prakash A., Lynn A.M. (2020). Structure-based virtual screening, molecular dynamics simulation and MM-PBSA toward identifying the inhibitors for two-component regulatory system protein NarL of Mycobacterium Tuberculosis. J. Biomol. Struct. Dyn..

