# Supplementary material for: Dieckol and Its Derivatives as Potential Inhibitors of SARS-CoV-2 Spike Protein (UK Strain: VUI 202012/01): A Computational Study
Source: Mar Drugs. 2021 Apr 25;19(5):242. doi: 10.3390/md19050242 (PMC8145291; doi:10.3390/md19050242)
Supplement: Supplementary file 1 [file marinedrugs-19-00242-s001.zip › marinedrugs-1136800-supplementary.pdf]

Supplementary Data

Discovery of high-affinity SARS-CoV-2 (UK strain: VUI 202012/01) Spike protein inhibitors from marine algae database using computational approaches

Mohammad Aatif[1*], Ghazala Muteeb[2], Abdulrahman Alsultan[3],

Adil Alshoaibi[4] and Bachir Yahia Khelif[1]

[1]Department of Public Health, College of Applied Medical Science, King Faisal University, Al-Ahsa 31982, Saudi Arabia

[2]Department of Nursing, College of Applied Medical Science, King Faisal University, Al-Ahsa 31982, Saudi Arabia

[3]College of Applied Medical Science, King Faisal University, Al-Ahsa 31982, Saudi Arabia

[4]Department of Physics, College of Science, King Faisal University, Al-Ahsa 31982, Saudi Arabia

Running Title: Marine-derived inhibitors of SARS-CoV-2 Spike protein

***Corresponding author**

Dr. Mohammad Aatif
(Corresponding author)
Department of Public Health
College of Applied Medical Science
King Faisal University
Al-Ahsa 31982
Saudi Arabia
Phone: +966560744262
E-mail: maahmad@kfu.edu.sa

Supplementary Table S1: Standard-precision (SP) molecular docking of selected ligands (having ≤ -4.000 kcal mol^{-1} in HTVS) against RBD of spike protein

S. No.	Compound ID	Docking score (kcal mol^{-1})	Glide g-score (kcal mol^{-1})	Glide e-model (kcal mol^{-1})	Glide energy (kcal mol^{-1})
1.	GA007	-6.951	-6.951	-85.102	-66.702
2.	GA004	-6.936	-6.936	-84.334	-58.483
3.	GA005	-6.685	-6.685	-69.120	-54.462
4.	GA006	-6.348	-6.348	-70.391	-58.024
5.	BE011	-5.098	-5.390	-62.335	-46.496
6.	BU002	-4.750	-4.750	-29.629	-23.116
7.	RL392	-4.665	-4.665	-25.053	-19.800
8.	RL425	-4.614	-4.614	-26.187	-20.572
9.	RP008	-4.613	-4.826	-24.097	-17.866
10.	BS007	-4.600	-4.600	-36.266	-29.534
11.	BE006	-4.599	-4.642	-21.174	-16.831
12.	BE012	-4.499	-4.645	-59.140	-43.493
13.	RR025	-4.456	-4.585	-37.792	-29.504
14.	RL249	-4.452	-4.452	-20.063	-17.600
15.	RP011	-4.423	-4.496	-30.088	-23.769
16.	GA010	-4.417	-4.491	-29.604	-23.164
17.	BU005	-4.405	-4.405	-23.387	-18.777
18.	RP063	-4.391	-4.391	-20.644	-16.329
19.	GA011	-4.371	-5.132	-54.028	-43.042
20.	RP017	-4.365	-4.766	-32.479	-25.734
21.	BS017	-4.353	-4.366	-55.937	-46.160
22.	RR040	-4.341	-4.742	-32.352	-25.620
23.	RJ007	-4.334	-4.334	-38.051	-33.370
24.	RL462	-4.307	-4.307	-31.220	-25.248
25.	BE013	-4.272	-4.410	-39.983	-30.576
26.	BS008	-4.269	-4.269	-28.960	-24.405
27.	RJ006	-4.232	-4.232	-36.463	-32.435
28.	RR065	-4.225	-4.230	-31.801	-23.120
29.	RL343	-4.181	-4.181	-30.186	-24.726
30.	RR052	-4.178	-4.632	-40.784	-32.553
31.	RL280	-4.176	-4.177	-25.038	-20.233
32.	RP004	-4.176	-4.435	-25.665	-18.352
33.	RL391	-4.175	-4.175	-21.782	-17.367
34.	RL077	-4.163	-4.163	-24.610	-19.739
35.	BE008	-4.119	-4.811	-53.817	-40.410
36.	RR066	-4.115	-4.135	-32.503	-26.324

37.	BS005	-4.105	-4.105	-22.296	-21.748
38.	RP005	-4.104	-4.161	-27.309	-22.388
39.	RL461	-4.101	-4.101	-28.001	-22.982
40.	BL017	-4.086	-4.165	-30.856	-21.581
41.	RR007	-4.076	-4.082	-31.196	-23.214
42.	BS009	-4.070	-4.070	-26.925	-27.120
43.	RR012	-4.070	-4.114	-34.223	-26.048
44.	BS006	-4.069	-4.069	-29.064	-25.549
45.	RR008	-4.064	-4.084	-32.471	-26.373
46.	RG013	-4.050	-4.050	-34.566	-28.144
47.	RP009	-4.044	-4.085	-27.167	-19.822
48.	BP003	-4.040	-4.040	-34.064	-27.935
49.	RR055	-4.032	-4.043	-26.118	-20.382
50.	BL014	-4.025	-4.191	-26.492	-18.503
51.	RL002	-4.021	-4.024	-29.147	-23.600

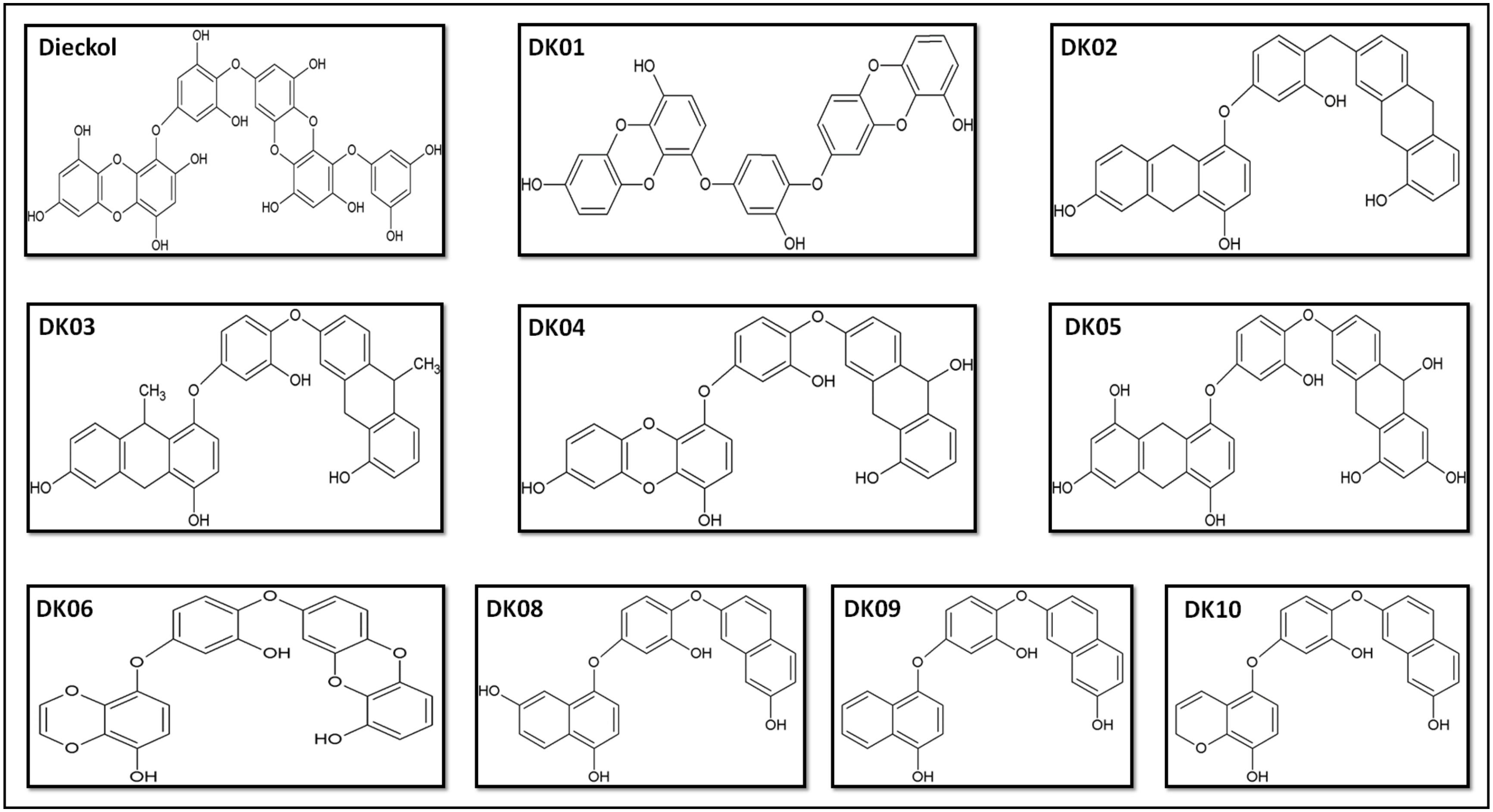

Supplementary Figure S1: Two-dimensional structures of Dieckol and its derivatives (DK01-DK10) used in the study.
